# Establishment and characterization of immortalized sweat gland myoepithelial cells

**DOI:** 10.1038/s41598-021-03991-5

**Published:** 2022-01-07

**Authors:** Tomohisa Hayakawa, Fumitaka Fujita, Fumihiro Okada, Kiyotoshi Sekiguchi

**Affiliations:** 1 Laboratory of Advanced Cosmetic Science, Graduate School of Pharmaceutical Sciences, Osaka University, 1-6 Yamadaoka, Suita, Osaka 565-0871 Japan; 2 Fundamental Research Institute, Mandom Corporation, Osaka, Japan; 3 Division of Matrixome Research and Application, Institute for Protein Research, Osaka University, 3-2 Yamadaoka, Suita, Osaka 565-0871 Japan

**Keywords:** Cytological techniques, Cell culture, Tissue culture, Senescence, Self-renewal, Skin stem cells, Skin diseases, Cells

## Abstract

Sweat glands play an important role in thermoregulation via sweating, and protect human vitals. The reduction in sweating may increase the incidence of hyperthermia. Myoepithelial cells in sweat glands exhibit stemness characteristics and play a major role in sweat gland homeostasis and sweating processes. Previously, we successfully passaged primary myoepithelial cells in spheroid culture systems; however, they could not be maintained for long under in vitro conditions. No myoepithelial cell line has been established to date. In this study, we transduced two immortalizing genes into primary myoepithelial cells and developed a myoepithelial cell line. When compared with primary sweat gland cells, the immortalized myoepithelial cells (designated "iEM") continued to form spheroids after the 4th passage and expressed α-smooth muscle actin and other proteins that characterize myoepithelial cells. Furthermore, treatment with small compounds targeting the Wnt signaling pathways induced differentiation of iEM cells into luminal cells. Thus, we successfully developed an immortalized myoepithelial cell line having differentiation potential. As animal models are not useful for studying human sweat glands, our cell line will be helpful for studying the mechanisms underlying the pathophysiology of sweating disorders.

## Introduction

The eccrine sweat gland, an exocrine gland in the skin, plays a pivotal role in the thermoregulation of the human body through sweat secretion. The eccrine sweat gland is a single tube composed of a secretory coil and a duct tube. The secretory coil consists of myoepithelial and secretory luminal cells, and plays a major role in the production and delivery of sweat to the skin surface^1^. Myoepithelial cells have been hypothesized to regulate sweat secretion through the contraction of secretory coils^2^, whereas secretory luminal cells release sweat into the lumen of secretory coils^3^. Myoepithelial cells in secretory coils play a pivotal role in maintaining sweat glands through self-renewal and differentiation into luminal cells^4,5^.

Myoepithelial cells highly express α-smooth muscle actin (SMA) that contributes to the generation of contractile force during sweat secretion^2^. This contraction is thought to be induced by acetylcholine via cholinergic receptor M3, which is highly expressed in myoepithelial cells^3^. Myoepithelial cells express the integrin α6β1 and keratin 14 (K14) genes, which are also expressed in the stem cells of the interfollicular epidermis and mammary gland. However, myoepithelial cells do not express keratin 8 (K8) and 18 (K18), which are expressed in secretory luminal cells^5^.

To further understand the physiology of sweat glands, long-term in vitro culturing of myoepithelial cells is a promising model. However, myoepithelial cells cannot be maintained in a two-dimensional monolayer culture system; only a spheroid culture system without cell adhesion can maintain myoepithelial cells up to the third passage^5,6^. Mammary gland cells, another type of well-studied exocrine gland cells, also require a spheroid suspension culture system to preserve their stemness characteristics, although these cells fail to form spheroids after the fourth passage^7^. This limitation was circumvented using immortalized mammary gland cells by transduction of two immortalizing genes such as human telomerase reverse transcriptase (hTERT) and the Simian virus 40 large T and small t antigen (SV40Tt), which can be maintained in a spheroid culture system beyond the fourth passage^8^.

In the present study, we aimed to establish immortalized myoepithelial cells in a spheroid culture system using the same approach. We found that the spheroid culture system could not maintain myoepithelial cells after the third passage. Therefore, we established immortalized myoepithelial cells overexpressing hTERT and SV40Tt. These immortalized cells retained myoepithelial cell phenotypes, including their ability to differentiate into luminal cells.

## Results

### Limited culture period for primary sweat gland cells

**Figure 1:**
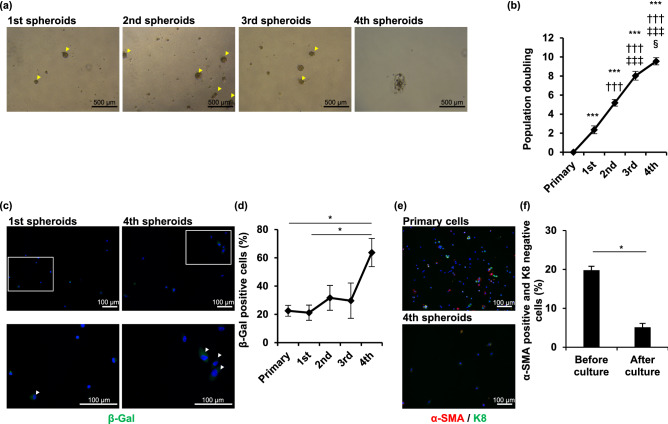
Serially passaged primary sweat gland cells undergo spheroid formation arrest. (**a**) Each image shows sweat gland cell spheroids from the 1st to 4th generation. The cells were cultured in suspension and passaged every seven days. Arrowheads indicate spheroids. (**b**) Growth curves of sweat gland cells derived from five different patients in each generation. Data are presented as the mean ± standard error (S.E.) of five independent experiments (n = 5). ^§^P < 0.05; ***, ^†††^, ^‡‡‡^, P < 0.001 (*,**versus primary, †† versus 1st spheroids, ‡‡ versus 2nd spheroids, § versus 3rd spheroids). (**c**) Images show β-galactosidase (β-gal) staining of cells at the 1st and 4th generation of spheroids. Arrowheads indicate β-gal-positive cells. The bottom panels show magnified views of the areas shown in the upper panels. Nuclei were stained with 4′,6-diamidino-2-phenylindole (DAPI). (**d**) Rate of β-gal-positive cells derived from spheroids of each generation. At least 50 cells were counted in each experiment. Data are presented as the mean ± S.E. of five independent experiments (n = 5). *P < 0.05. (**e**) Immunofluorescence staining of cells derived from sweat gland tissues (primary cells) and 4th generation spheroids (4th spheroids) for α-smooth muscle actin (α-SMA) (red) and keratin 8 (K8) (green). Nuclei were stained with DAPI. (**f**) Rate of α-SMA-positive and K8-negative cells before and after the culture until spheroid formation arrest. At least 50 cells were counted in each experiment. Data are presented as the mean ± S.E. of five independent experiments (n = 5). * P < 0.05. Scale bars: 500 μm in A and 100 μm in b and c.

To assess the long-term spheroid formation ability of human sweat gland cells in vitro, we first performed serial passage culture of sweat gland cells under spheroid suspension conditions. Consistent with a previous report^5^, sweat gland cells formed spheroids and proliferated until the third passage, but the cell aggregates shrank and became nonspherical thereafter (Fig. 1a,b and Supplementary Fig. S1a). Because cell proliferation arrest is commonly caused by the induction of cellular senescence, a state of irreversible cell cycle arrest in cultured cells, we assessed the accumulation of the senescent cells in the serial passage of sweat gland cells. The number of β-gal-positive sweat gland cells at the fourth passage was significantly higher than that at the first passage and before the spheroid culture (*P* = 0.018 and 0.022) (Fig. 1c,d and Supplementary Fig. S1b). We have previously reported that myoepithelial cells are stem cells in sweat glands, which proliferate and form spheroids^5,6^. To determine the proportion of myoepithelial cells in total sweat gland cells, we examined the number of α-SMA-positive cells in the primary sweat gland cells and those arrested in spheroid formation. The proportion of α-SMA-expressing cells significantly decreased when the cells failed to form spheroids (*P* = 0.023) (Fig. 1e,f). These results indicated that the in vitro spheroid culture of human sweat gland cells cannot be maintained for more than the fourth passage under spheroid suspension conditions. Moreover, the lack of cell proliferation and spheroid formation might be caused by a reduction in the proliferative ability of myoepithelial stem cells after serial passages in spheroid cultures.

### Introduction of immortalizing genes prolongs the cell culture period in sweat glands

**Figure 2:**
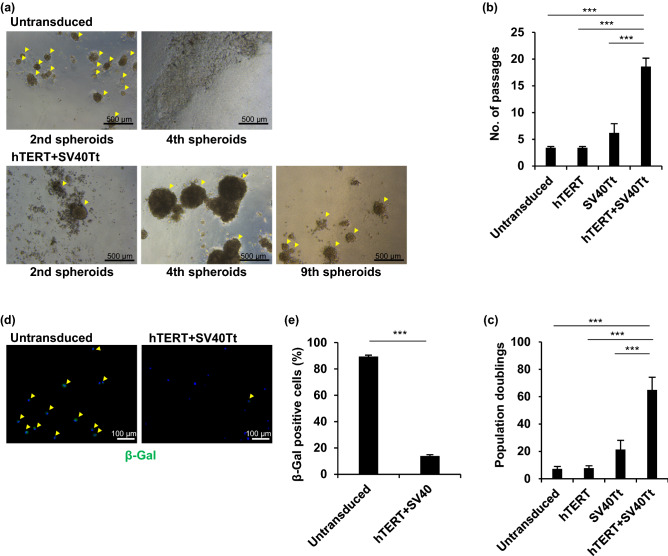
Establishment of immortalized sweat gland cells in spheroid culture. (**a**) Spheroids formed from sweat gland cells transduced with human telomerase reverse transcriptase (hTERT) and Simian Virus 40 large T and small t antigen (SV40Tt) genes at the indicated generation of spheroids. The cells were passaged once daily for seven days. Arrowheads indicate spheroids. (**b**,**c**) Number of passages (**b**) and population doublings (**c**) in spheroid culture of sweat gland cells transduced with hTERT and/or SV40Tt genes. Data are presented as the mean ± standard error (S.E.) of five independent experiments (n = 5). ***P < 0.001. (**d**) β-galactosidase (β-gal) staining of sweat gland cells transduced with hTERT + SV40Tt and untransduced cells at the 4th spheroid. Arrowheads indicate β-gal-positive cells. Nuclei were stained with 4′,6-diamidino-2-phenylindole (DAPI). (**e**) Rate of β-gal-positive cells in hTERT + SV40Tt transduced cells and untransduced cells. At least 50 cells were counted under each condition. Data are presented as the mean ± S.E. of five independent experiments (n = 5). ***P < 0.001. Scale bars: 500 μm in A and 100 μm in d.

As the growth of sweat gland cells under in vitro culture was disturbed by spontaneous cellular senescence, primary sweat gland cells could not be cultured for a long time. According to a previous study on glandular cell immortalization^8^, we attempted to immortalize sweat gland cells by the transduction of the hTERT and SV40Tt genes (Fig. S2). The hTERT- and SV40Tt-transduced cells displayed spheroid formation at the fourth and ninth passages (Fig. 2a), and were competent in forming spheroids even after the 19th passage, on average (Fig. 2b). Population doubling was also significantly increased in the hTERT- and SV40Tt-transduced cells, capable of population doubling 65 times compared with 7 times in untransduced cells (*P* < 0.001) (Fig. 2c). In contrast, spheroid formation and population doubling in single gene-transduced cells were not significantly different from those in the untransduced cells. The hTERT- and SV40Tt-transduced cells formed a spherical structure until day 4 and the spheroids size continued to increase thereafter (Supplementary Fig. S3). Furthermore, the cryopreserved hTERT-and SV40Tt-transduced cells were capable of forming spheroids and competent in passaging several times (Supplementary Fig. S4a). Moreover, the proportion of β-gal-positive senescent cells in hTERT- and SV40Tt-transduced cells was significantly lower than that in the untransduced control cells at the fourth or fifth passage when these cells failed to form spheroids (*P* < 0.001) (Fig. 2d,e). This indicated that we successfully generated immortalized sweat gland cells in the suspension spheroid culture system.

### Immortalized sweat gland cells maintain myoepithelial cell properties

**Figure 3:**
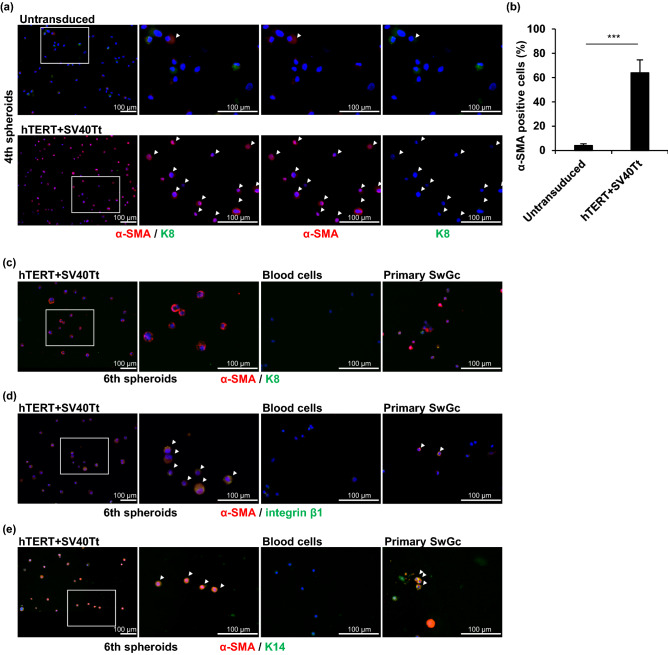
Immortalized sweat gland cells exhibit myoepithelial cell properties. (**a**) Immunofluorescence of human telomerase reverse transcriptase and Simian Virus 40 large T and small t antigen (hTERT + SV40Tt) gene-transduced cells and untransduced cells in the 4th spheroids for α-smooth muscle actin (α-SMA) and keratin 8 (K8). (**b**) Rate of α-SMA-positive and K8-negative cells in hTERT + SV40Tt gene-transduced cells and untransduced cells at the 4th spheroids. At least 50 cells were counted under each condition. Data are presented as the mean ± S.E. of five independent experiments (n = 5). ***P < 0.001. (**c**–**e**) Immunofluorescence of hTERT + SV40Tt-transduced cells in the sixth spheroid, blood cells (negative control), and primary sweat gland cells (primary sweat gland cells; positive control) for α-SMA and K8 (**c**), α-SMA and integrin β1 (**d**), and α-SMA and keratin 14 (K14) (**e**). Arrowheads indicate α-SMA-positive cells (**a**), α-SMA and integrin β1 positive cells (**d**), and α-SMA and K14 positive cells (**e**). The second panels from the left are magnified views of the boxed areas in the left panels. Scale bars: 100 μm.

As immortalized sweat gland cells were competent in spheroid formation, which was also observed in the culture of myoepithelial cells^5^, we examined whether the immortalized cells expressed myoepithelial cell-specific protein. α-SMA-positive cells were predominantly observed in the immortalized cells at the fourth passage, whereas α-SMA-positive cells were hardly observed in the untransduced control cells (*P* < 0.001) (Fig. 3a,b). The immortalized cells retained α-SMA-positive cells after further passages (e.g., at the sixth passage) and did not produce K8, a luminal cell marker (Fig. 3c). Immortalized cells retained α-SMA production even after cryopreservation (Supplementary Fig. S4b). Moreover, the majority of immortalized sweat gland cells at the sixth passage indicated the presence of α-SMA with integrin β1 or K14, similar to those in the primary myoepithelial cells (Fig. 3d,e). These results strongly indicated that our sweat-gland-cell-derived immortalized cells retained myoepithelial-cell-specific protein production. Therefore, we termed the generated cells “immortalized eccrine sweat gland myoepithelial (iEM) cells.”

### iEM cells can differentiate into luminal cells

**Figure 4:**
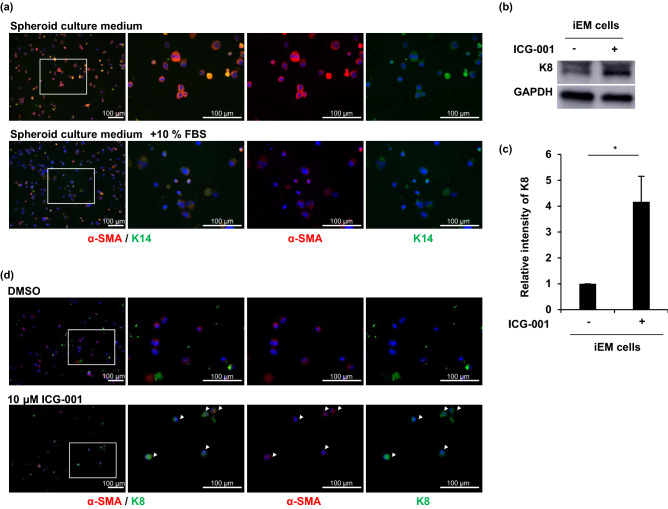
Immortalized eccrine sweat gland myoepithelial cells retain the potential of differentiation into luminal cells. (**a**) Immunofluorescence of immortalized myoepithelial (iEM) cells cultured with or without 10% fetal bovine serum (FBS) for α-smooth muscle actin (α-SMA) and keratin 14 (K14) for seven days. The second panels from the left are magnified views of the boxed areas in the left panels. Nuclei were stained with 4′,6-diamidino-2-phenylindole (DAPI). (**b**) Immunoblots of iEM cells treated with 10 μM ICG-001 (right) and dimethylsulfoxide (DMSO) (left) for keratin 8 (K8) and glyceraldehyde-3-phsphate dehydrogenase (GAPDH). (**c**) Relative quantitative values of K8 gene expression normalized to GAPDH gene expression. Quantification was performed using ImageJ software (version 1.53, https://imagej.nih.gov/ij/). Data are presented as the mean ± standard error (S.E.) of five independent experiments (n = 5). * P < 0.05. (**d**) Immunofluorescence for α-SMA (red) and K8 (green) of iEM cells cultured with 10 μM ICG-001 and DMSO for seven days. Arrowheads indicate K8-positive cells. The second panels from the left are magnified views of the boxed areas in the left panels. Scale bars: 100 μm.

To confirm whether iEM cells are capable of differentiation, similar to primary myoepithelial cells, we cultured iEM cells in serum-containing spheroid culture medium used for inducing differentiation of glandular stem cells in the mammary and meibomian glands^7,9^. Serum-supplemented culture medium reduced α-SMA and K14 production in iEM cells, whereas serum-free culture medium did not (Fig. 4a). Additionally, adherent culture conditions, which induced the loss of myoepithelial cells in primary sweat gland cells, also reduced α-SMA and K14 gene expression in iEM cells (Supplementary Fig. S5a). Thus, iEM cells were responsive to serum as the cell differentiation stimulus, which was identical to mammary gland stem cells^8^. However, this culture medium-containing serum did not show clear differentiation of iEM cells into luminal cells, and K8 expression was not detected (Supplementary Fig. S5b). Therefore, we attempted to find a suitable stimulation for the differentiation of iEM cells into luminal cells. We screened small compounds capable of differentiating iEM cells into luminal cells from a panel of small compounds (differentiation inhibitors and activators), by employing K18 production as the marker for luminal cell production. Thirty-four compounds were found to increase K18 mRNA expression in iEM cells (Table 1). Among these, ICG-001, a Wnt signaling pathway inhibitor, increased the production of K8, another luminal-cell-specific protein, in iEM cells (Fig. 4b,c). We performed immunofluorescence staining to assess K8 and α-SMA protein production in ICG-001-treated iEM cells. K8-producing cells were found in iEM cells treated with ICG-001 but not in the control cells (Fig. 4d). These results indicated that iEM cells are competent in differentiating into luminal cells when small compounds, including ICG-001, are provided. Therefore, iEM cells possess myoepithelial cell characteristics, including specific protein expression and differentiation potential, similar to primary myoepithelial cells.

**Table 1:** Overview of K18 mRNA expression in iEM cells treated with the indicated compounds.

	Compounds	Target signaling	K18 induction
1	APTSTAT3-9R	STAT	+
2	IWP-L6	Wnt/β-catenin	+
3	IC261	Casein Kinase	+
4	Oclacitinib maleate	JAK	+
5	MK-4101	Hedgehog/Smoothened	+
6	SKL2001	Wnt/β-catenin	+
7	ICG-001	Wnt/β-catenin	+
8	IM-12	GSK-3	+
9	β-Glycerophosphate	phosphatase	+
10	FH535	PPAR, Wnt/β-catenin	+
11	XMD8-92	ERK	+
12	Theophylline	TGF-β/Smad	+
13	LDN-193189 2HCl	TGF-β/Smad	+
14	LF3	Wnt/β-catenin	+
15	Pacritinib	FLT3, JAK	+
16	KYA1797K	Wnt/β-catenin	+
17	MK-0752	Beta Amyloid	+
18	Wnt agonist 1	Wnt/β-catenin	+
19	RO4929097	Beta Amyloid	+
20	Taladegib	Hedgehog/Smoothened	+
21	XL019	JAK	+
22	SB415286	GSK-3	+
23	HO-3867	STAT	+
24	Astragaloside A	Calcium Channel	+
25	GANT61	Gli	+
26	Filgotinib	JAK	+
27	AZD2858	GSK-3	+
28	PRI-724	Wnt/β-catenin	+
29	SB431542	TGF-β/Smad	+
30	RepSox	TGF-β/Smad	+
31	GSK429286A	ROCK	+
32	KY02111	Wnt/β-catenin	+
33	CP21R7 (CP21)	Wnt/β-catenin	+
34	Indirubin	GSK-3	+
35	Fedratinib	JAK	0
36	PD173955	Bcr-Abl	0
Control	DMSO		0
37	Galunisertib	TGF-β/Smad	0
38	O4I2	Oct3/4	0
Negative	blood cells		0

Immortalized myoepithelial (iEM) cells under each condition were cultured for 7 days. Gene expression was measured using reverse transcriptase polymerase chain reaction (RT-PCR) using 18S rRNA as an internal reference.

+: increased (> two fold).

0: no difference.

## Discussion

Sweat gland homeostasis is thought to be maintained by myoepithelial cells and sweat gland stem cells^4,5^. To control self-renewal and differentiation of stem cells, understanding the molecular mechanisms underlying stem cell differentiation is important. To study the molecular mechanisms underlying the differentiation of sweat gland myoepithelial cells in vitro, myoepithelial cells that can stably proliferate in a culture system are required. In our study, the primary sweat gland cells showed spheroid hypoplasia and attenuation of cell proliferation after the third passage. Therefore, we established iEM cells for the molecular analysis of sweat gland stem cells. iEM cells, transduced with hTERT and SV40Tt genes, continued to form spheroids after the 4th passage and expressed myoepithelial cell markers. Spheroids of iEM cells continued to increase in size after formation, suggesting that iEM cells are resistant to contact inhibition, as is the case with other cells transduced with hTERT and SV40Tt. iEM cells formed spheroids until the 19th passage. Moreover, the number of iEM cells was similar to or less than that of seeded cells at the last passage, indicating that most of them might enter the senescence phase as normal fibroblasts. Thus, iEM cells might be considered an in vitro model for investigating the functions of myoepithelial cells in eccrine sweat glands.

Previously, Lee and Dessi reported an epithelial cell line established from human sweat glands following the infection with simian virus 40^10^. The cell line, NCL-SG3, proliferates as an epithelial monolayer and expresses PAR-2 and TMEM16A genes, which are highly expressed in luminal cells but not in myoepithelial cells^11,12^. NCL-SG3 cells also exhibit an increased rate of potassium efflux following ionomycin treatment^13^. The potassium channels activated by intracellular calcium are expressed in luminal cells in secretory coils but not in ductal cells^14^, indicating that NCL-SG3 cells are reminiscent of luminal cells of the secretory coils. Supporting this observation, the expression of cystic fibrosis transmembrane regulator, which is abundantly found in ductal cells^15^, is not observed in NCL-SG3^16^. Thus, although established from human sweat gland cells, NCL-SG3 cannot be used as an in vitro model for myoepithelial cells of the secretory coils. There are no other cell lines derived from human sweat glands that express myoepithelial cell phenotypes; hence, the iEM cells developed in our study are the only immortalized cells suitable for an in vitro model of myoepithelial cells in sweat glands.

The iEM cells showed limited passaging numbers (approximately 19) and population doubling (approximately 65), as was the case with other immortalized epithelial cells reported previously. Immortalized mammary gland cells^8^ and keratinocytes^17^ showed a population doubling of 100 and 108, respectively. Immortalized mammary gland cells were used as an experimental model for the gene function analysis of breast cancer cells^18^. Therefore, iEM cells are a promising resource for studying the molecular and genetic characteristics of the myoepithelial cells of sweat glands. However, iEM cells cannot be maintained in a two-dimensional monolayer culture (Supplementary Fig. S4). Such limitations in 2D culture may possibly be imposed by the immortalization protocol, as Zhao et al*.* reported that mammary gland cells immortalized by the introduction of dominant active RhoA grow beyond 300 population doublings, which is higher than that immortalized using hTERT and SV40Tt^19^. Other protocols for immortalization such as introduction of HPV-E6/E7, c-myc, and RhoA may improve the proliferation potential and protein production of immortalized myoepithelial cells that can be maintained in a monolayer culture^19–21^. However, additional studies are needed to determine the suitable immortalization conditions for the long-term culture of myoepithelial cells.

Hyperhidrosis, hypohidrosis, and heatstroke are sweat gland-related diseases. Therapeutic strategies against hyperhidrosis (e.g., Botox injection and anticholinergic drugs) have been established to elucidate the mechanisms controlling sweat secretion by the central nervous system^22^. However, therapeutic strategies against hypohidrosis and heatstroke, both of which are caused by sweat gland dysfunction, have not been well established^23,24^. To retain sweat gland function, myoepithelial cells behave as stem cells in sweat glands^4,5^. As a stable culture system for myoepithelial cells has not been established, and approaches to elucidate the functions of myoepithelial cell maintenance are limited^25^. However, iEM cells can stably proliferate in a spheroid culture system, express myoepithelial cell-specific proteins, and exhibit differentiation potential. Thus, iEM cells can serve as experimental tools to study the pathogenesis of hypohidrosis and heatstroke.

The incidence of heatstroke increases in elderly people because sweating rates decrease with aging^26–28^. Sweat gland atrophy with aging is one of the triggers for the reduction of sweat secretion^29,30^. As the exhaustion of stem cells causes organ atrophy with aging^31^, the dysfunction or decrease of myoepithelial cells in sweat glands with aging might lead to sweat gland atrophy. Comparing the function of iEM cells established from the sweat glands of elderly people with those from younger people might elucidate the mechanisms of reduction in cellular functions with aging. Moreover, such additional efforts using iEM cells will be effective in finding a therapeutic method for hypohidrosis and heatstroke. As skin burns are repaired without sweat glands^32^, such patients suffer from reduced quality of life (QOL) because of the impaired thermoregulation. Technology for enabling regenerative skin with sweat glands will improve the QOL of burn patients. The iEM cells developed in our study are promising resources for the development of regenerative skin with sweat glands. Moreover, as common experimental animals (e.g., mice, rats, and more) do not have eccrine sweat glands for thermoregulation, such animal models are limited in the study of the physiological functions of human eccrine sweat glands. Since iEM cells preserve the in vivo-like characters of myoepithelial cells, they may compensate for the deficiency of in vivo models.

## Materials and methods

### Human skin tissues

Human skin tissues were obtained with informed consent from Kinugasa Clinic (Osaka, Japan) and Bizcom Japan (Tokyo, Japan). Experiments using human skin were approved by the ethics committee of Osaka University. All research was performed in accordance with relevant guidelines and regulations. Informed consent was obtained from all participants.

### Sweat gland isolation and spheroid cell culture

Sweat glands were obtained by performing skin biopsies using tweezers and scissors and disaggregated with 600 U/mL collagenase type II (Worthington Biochemical Corporation, Lakewood, NJ) in Mammocult Human Medium (Stemcell Technologies, Vancouver, BC, Canada) for 4–16 h at 37 °C and 5% CO_2_ using a tube rotator (NISSIN, Tokyo, Japan) at 20 rpm. After the enzymatic reaction, sweat glands were isolated using micropipettes (P1000). The isolated sweat glands were then sequentially incubated with 0.5% trypsin–EDTA (Gibco, Waltham, MA, USA) and 5 U/mL dispase (Gibco) at 37 °C by pipetting to dissociate sweat gland cells. The cells were then filtered through a 40-μm filter (Falcon, Corning, NY), centrifuged at 350×*g* for 5 min at 4 °C, and washed three times with Dulbecco’s phosphate-buffered saline (Gibco). The washed cells were seeded in 24-well ultra-low attachment culture plates (Corning Inc., Corning, NY, USA) with spheroid culture medium (Supplementary Table S1) at a density of 2500 cells/well. Sweat gland spheroids were enzymatically dissociated into single cells and re-cultured under the same conditions. The spheroids were passaged once every seven days.

### Immunofluorescence staining

Cells were fixed with 4% paraformaldehyde for 15 min at 25 °C. Fixed cells were mounted on slides and incubated at 37 °C for 8–16 h for attachment of the cells to the slides. The cells fixed on the slides were permeabilized with 0.5% Triton X-100. The cells were then blocked with 3% bovine serum albumin (BSA) solution for 30 min and incubated with the primary antibody diluted in 3% BSA solution overnight (16 h) at 4 °C. Subsequently, the cells were washed with 0.05% Triton X-100 in phosphate-buffered saline (PBS-T) three times and incubated with the secondary antibody diluted in 1% BSA solution for 1.5 h at 25 °C. After incubation with the secondary antibody, the cells were washed with PBS-T three times and stained with 4′,6-diamidino-2-phenylindole (DAPI) for 5 min at 25 °C. After washing the slides with PBS, the cells were covered with glass slips. Fluorescence images were obtained using a BZ-X710 microscope (Keyence Japan, Osaka, Japan) at 20X magnification. The antibodies used are listed in Supplementary Table S2.

### Senescence-associated β-galactosidase (β-gal) activity assay

The cells fixed on slides were stained using SPiDER-βgal (Dojindo, Kumamoto, Japan), according to the manufacturer’s protocol. Then the cells were washed with PBS three times and stained with DAPI for 5 min at 25 °C. After washing the slides with PBS, the cells were covered with glass slips. Fluorescence images were obtained using a BZ-X710 microscope (Keyence Japan, Osaka, Japan) at × 20 magnification.

### Lentivirus infection

Lentivirus vectors carrying hTERT and SV40Tt genes were purchased from Applied Biological Materials (Richmond, BC, Canada). Sweat gland cells forming spheroids were immortalized by infection with viral medium (spheroid culture medium without Matrigel (BD Biosciences, San Jose, CA) and antibiotic–antimycotic (Gibco), 5 × 10^6^ U/mL lentivirus vectors, and 10 μg/mL polybrene (Santa Cruz Biotechnology, Santa Cruz, CA, USA)). The viral medium was replaced with fresh medium after 6 h.

### Quantitative reverse transcriptase polymerase chain reaction (RT-PCR) analysis

Total RNA was extracted using TRI-reagent (MRC, Cincinnati, OH) according to the manufacturer’s instructions, and complementary DNA (cDNA) was synthesized from RNA using the QuantiTect Reverse Transcription Kit (QIAGEN, Hilden, Germany). The synthesized cDNA was subject to real-time PCR analysis using the THUNDERBIRD SYBR qPCR Mix (TOYOBO, Osaka, Japan) and a ViiA7 real-time PCR system (Applied Biosystems, Foster City, CA, USA). The primer sequences used for PCR are listed in Supplementary Table S3.

### Agarose gel electrophoresis of PCR products

The cDNA obtained from total RNA was amplified by PCR using Veriti Thermal Cycler (Applied Biosystems). The amplified cDNA was subject to 2% agarose gel electrophoresis for 30 min using Midori Green (Nippon Genetics, Tokyo, Japan). DNA band images were obtained using Limited-STAGE (AMZ Systems Science, Osaka, Japan). The targeted bands were analyzed using the ImageJ software version 1.53 (NIH, Bethesda, MD, USA, http://imagej.nih.gov/ij/).

### Culture of immortalized myoepithelial cells with small compounds

Immortalized myoepithelial cells were seeded in 24-well ultra-low attachment culture plates (Corning) containing spheroid culture medium (10,000 cells/well). The cells were cultured with each compound in the library consisting of 99 small compounds, i.e., targeting factors related to stem cell maintenance and differentiation (Selleck Chemicals, Houston, TX, USA) (Supplementary Table S4) for seven days. K18 expression in the cultured cells was measured using RT-PCR.

### Western blot analysis

The cells were lysed in a solution (50 mM Tris–HCl [pH 7.5], 125 mM NaCl, 1.0% Nonidet P-40) and subjected to western blot analysis with 12%–polyacrylamide gel and polyvinylidene fluoride membrane. The antibodies used in western blot are listed in Supplementary Table S2. Target proteins were detected using ECL prime solution (GE Healthcare, Chicago, IL, USA) and Amersham Imager 600 (GE Healthcare). The targeted bands were analyzed using the ImageJ software version 1.53 (NIH, Bethesda, MD, USA).

### Statistical analysis

All data are represented as the mean ± standard error of the mean. Statistical significance was determined by the unpaired two-tailed Student’s t-test, Mann–Whitney U test, Steel–Dwass test, and Tukey–Kramer test using the Prism 7 software version 7.04 (GraphPad Software, San Diego, CA, https://www.graphpad.com/scientific-software/prism/). Differences were considered statistically significant at *P* < 0.05.

## Supplementary Information

Supplementary Figures.

Supplementary Tables.

## Data Availability

All data generated or analyzed during this study are included in this published article (and its Supplementary Information files).
