# Supplementary material for: Establishment and characterization of immortalized sweat gland myoepithelial cells
Source: Sci Rep. 2022 Jan 7;12:7. doi: 10.1038/s41598-021-03991-5 (PMC8741770; doi:10.1038/s41598-021-03991-5)
Supplement: Supplementary file 1 — Supplementary Figures. [file 41598_2021_3991_MOESM1_ESM.pdf]

Title: Establishment and characterization of immortalized sweat gland myoepithelial cells

Authors and affiliations: Tomohisa Hayakawa[1], Fumitaka Fujita[1,2,*], Fumihiro Okada[1,2], Kiyotoshi Sekiguchi[3,*]

[1]Laboratory of Advanced Cosmetic Science, Graduate School of Pharmaceutical Sciences, Osaka University, 1-6 Yamadaoka, Suita Osaka *565-0871*, Japan

[2]Fundamental Research Institute, Mandom Corporation, Osaka, Japan

[3]Division of Matrixome Research and Application, Institute for Protein Research, Osaka University, 3-2 Yamadaoka, Suita Osaka *565-0871*, Japan

* Corresponding author

E-mail: sekiguch@protein.osaka-u.ac.jp; fujita-f@phs.osaka-u.ac.jp

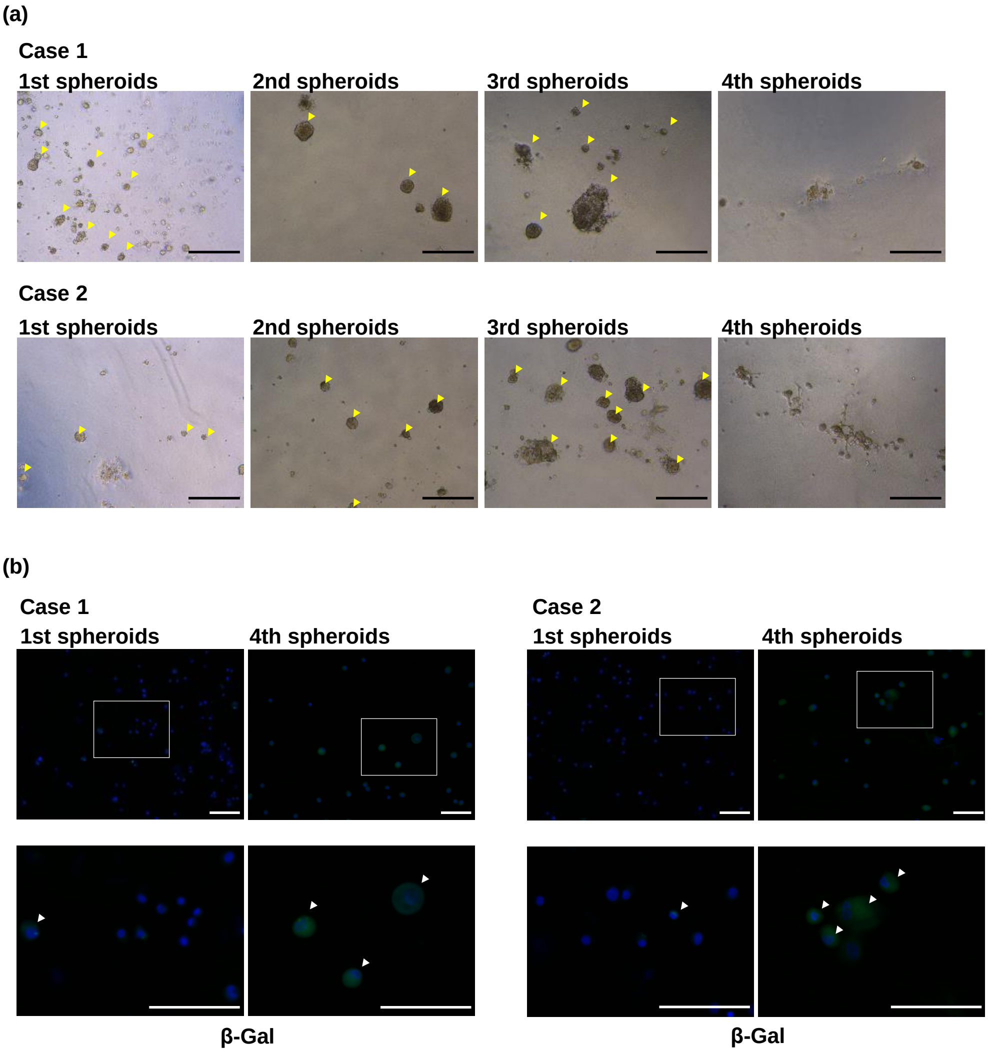

Supplementary Figure S1. Serially passaged primary sweat gland cells undergo spheroid formation arrest.
a: Images show sweat gland cell spheroids at the indicated generations. The cells were cultured in suspension and passaged once daily for 7 days. Sweat gland cells were obtained from the eyelid skin of a 65-year-old woman (Case 1; upper panels) and a 51-year-old woman (Case 2; bottom panels). Arrowheads indicate spheroids. b: Images show β-galactosidase (β-gal) staining of the cells derived from the indicated generations of Case 1 (left) and Case 2 (right) spheroids. The bottom panels show magnified views of the boxed areas in the upper panels. Arrowheads indicate β-gal-positive cells. Nuclei were counterstained with 4′,6-diamidino-2-phenylindole (DAPI). Scale bars: 500 μm in (a) and 100 μm in (b).

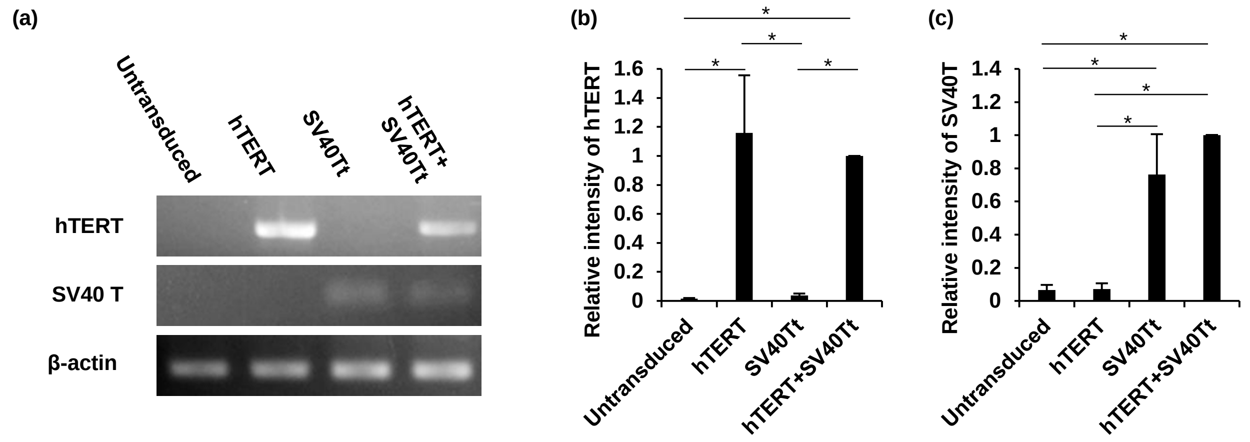

Supplementary Figure S2. Gene expression of human telomerase reverse transcriptase (hTERT) and Simian Virus 40 large T and small t antigen (SV40Tt) in cells transduced by hTERT, SV40 Tt, hTERT + SV40Tt, and untransduced cells.
a: Agarose gel electrophoresis of polymerase chain reaction (PCR)-amplified products of hTERT, SV40T, and β-actin on 2% agarose gel.
b–c: The graph shows the relative quantitative values of hTERT and SV40Tt expression normalized to those of β-actin. Quantification was performed using ImageJ software (version 1.53, https://imagej.nih.gov/ij/). Data are presented as the mean ± standard error (S.E.) of five independent experiments (n = 5). * $P < 0.05$; Steel-Dwass test.

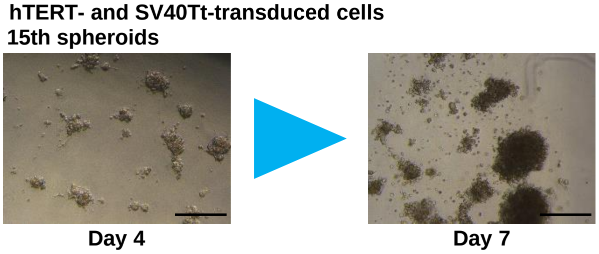

Supplementary Figure S3. Spheroids formed from hTERT- and SV40Tt-transduced cells continue to increase in size after spheroid formation.
Panels show spheroids formed from human telomerase reverse transcriptase and Simian Virus 40 large T and small t antigen (hTERT + SV40Tt)-transduced sweat gland cells. Scale bars: 500 µm.

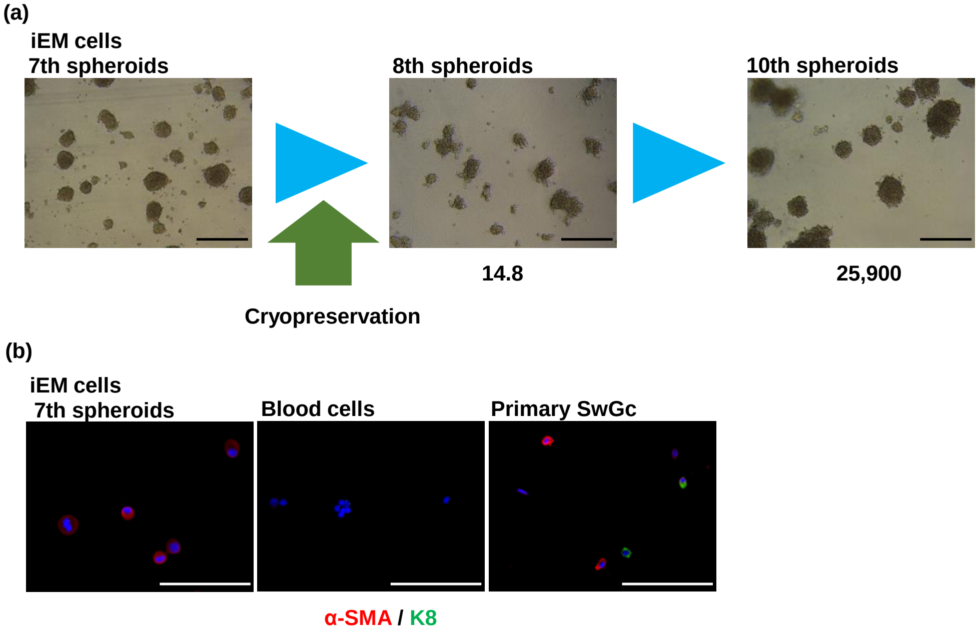

Supplementary Figure S4. Cryopreserved immortalized myoepithelial (iEM) cells maintain the proliferation ability, spheroid formation potential, and expression of α-smooth muscle actin (α-SMA).
a: Panels show spheroids formed from human telomerase reverse transcriptase and Simian Virus 40 large T and small t antigen (hTERT + SV40Tt)-transduced sweat gland cells. Bottom numbers represent the fold changes in cell number after the onset of cultivation of cryopreserved cells. b: Immunofluorescence staining of the cells derived from cryopreserved iEM cells in 7th spheroids (left), blood cells (negative control; center), and primary sweat gland cells (positive control; right) for α-SMA and keratin 8 (K8). Scale bars: 100 μm in (b) and 500 μm in (a).

(a)

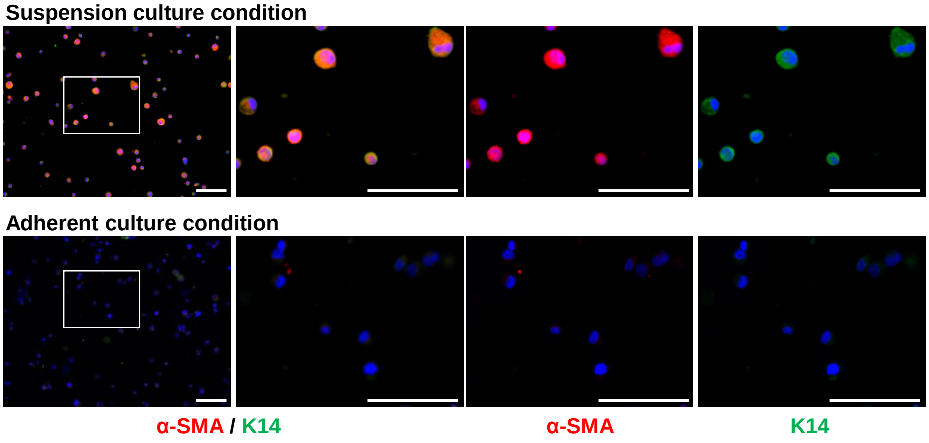

(b)

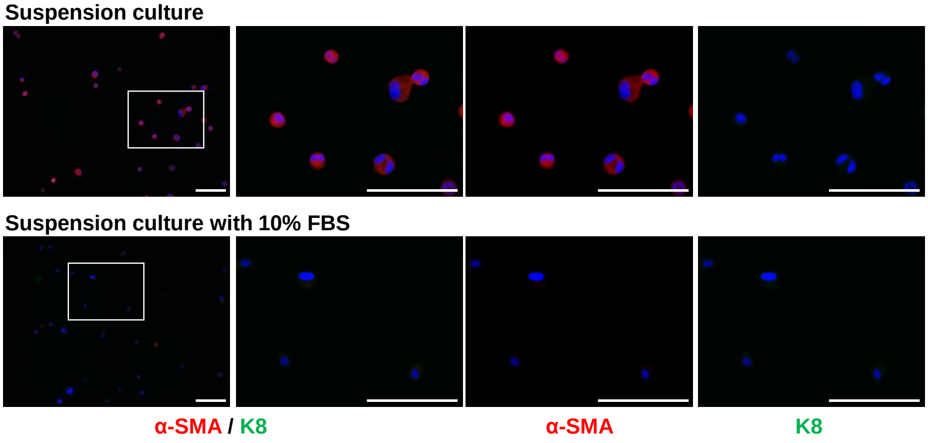

Supplementary Figure S5. Immortalized myoepithelial (iEM) cells cannot maintain their myoepithelial cell specific protein expression in adherent culture and fetal bovine serum (FBS)-containing culture conditions.
a: Immunofluorescence staining of iEM cells cultured under suspension or adherent conditions for α-smooth muscle actin (α-SMA) (red) and keratin 14 (K14) (green). b: Immunofluorescence staining of iEM cells cultured in suspension with or without FBS for α-SMA (red) and keratin 8 (K8) (green). The cells under each condition were cultured for seven days. The second from the left is a magnified view of the boxed areas in the left panels. Nuclei were counterstained with 4′,6-diamidino-2-phenylindole (DAPI). Scale bars: 100 μm.

(a)

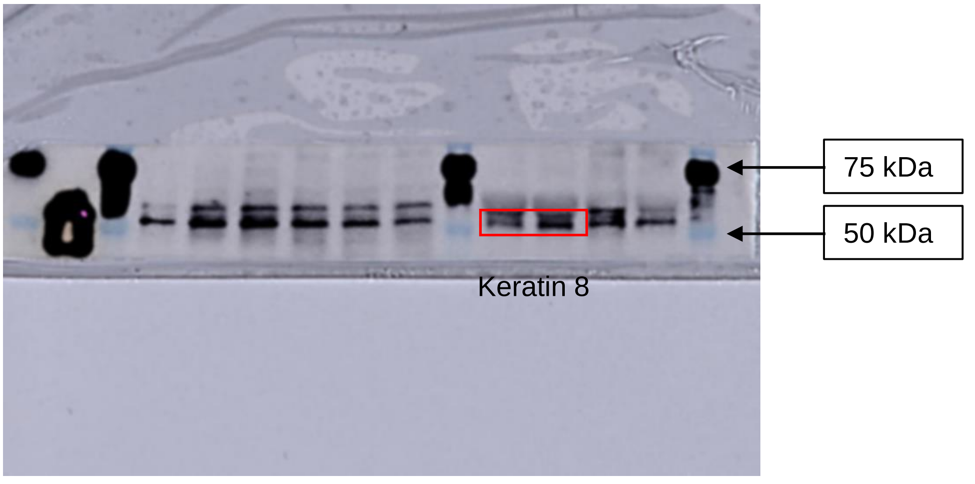

(b)

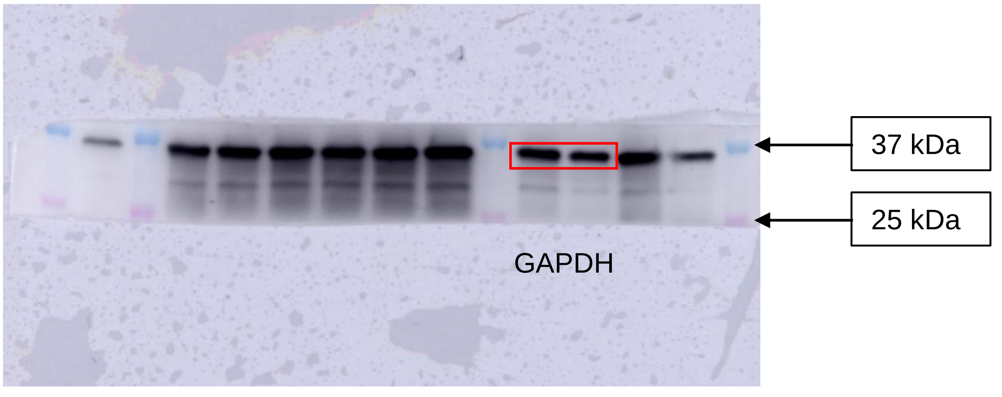

Supplementary Figure S6. Original images in Figure 4C.
Uncropped versions of figure images Keratin 8 (a) and GAPDH (b).

Note: To observe protein expression simultaneously, the membranes were cut before probing. All images in the Supplementary Figure file are the original, unprocessed versions of Figure 4 in the manuscript file and Supplementary Figure S2a. The indicated bands show the precise signal and target protein size.

(a)

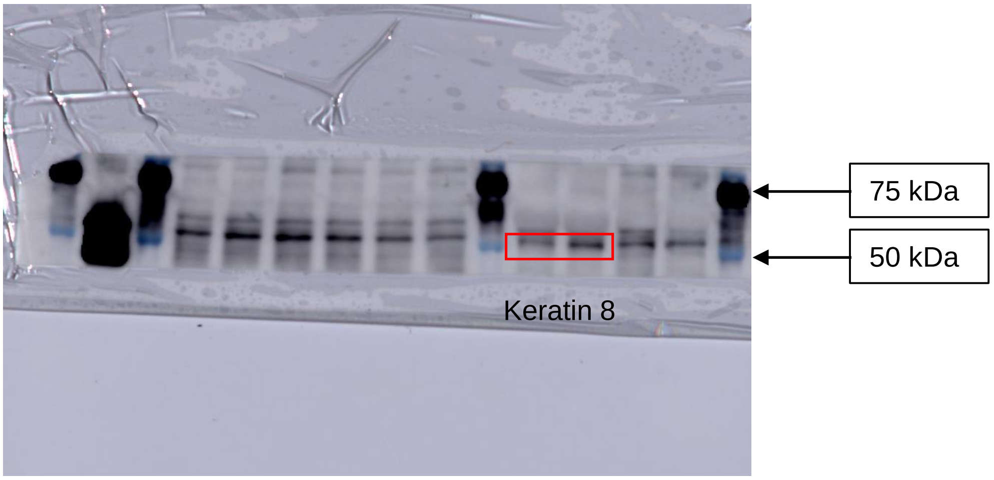

(b)

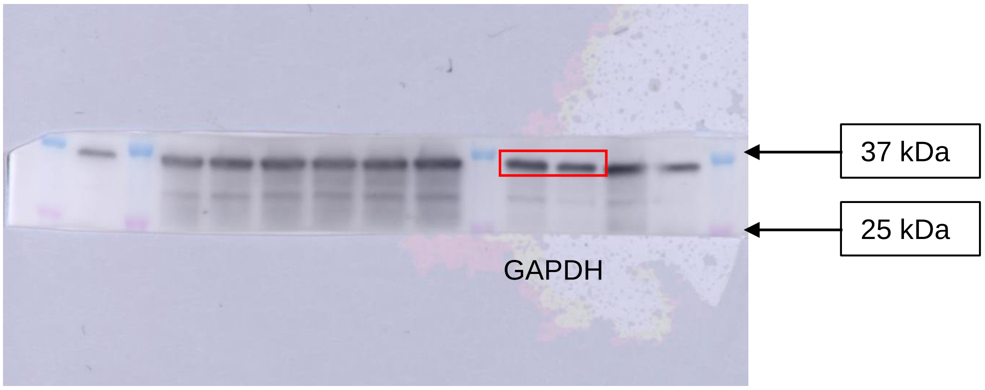

Supplementary Figure S7. Replicates of original images Figure 4C.
Uncropped versions of figure images Keratin 8 (a) and GAPDH (b).

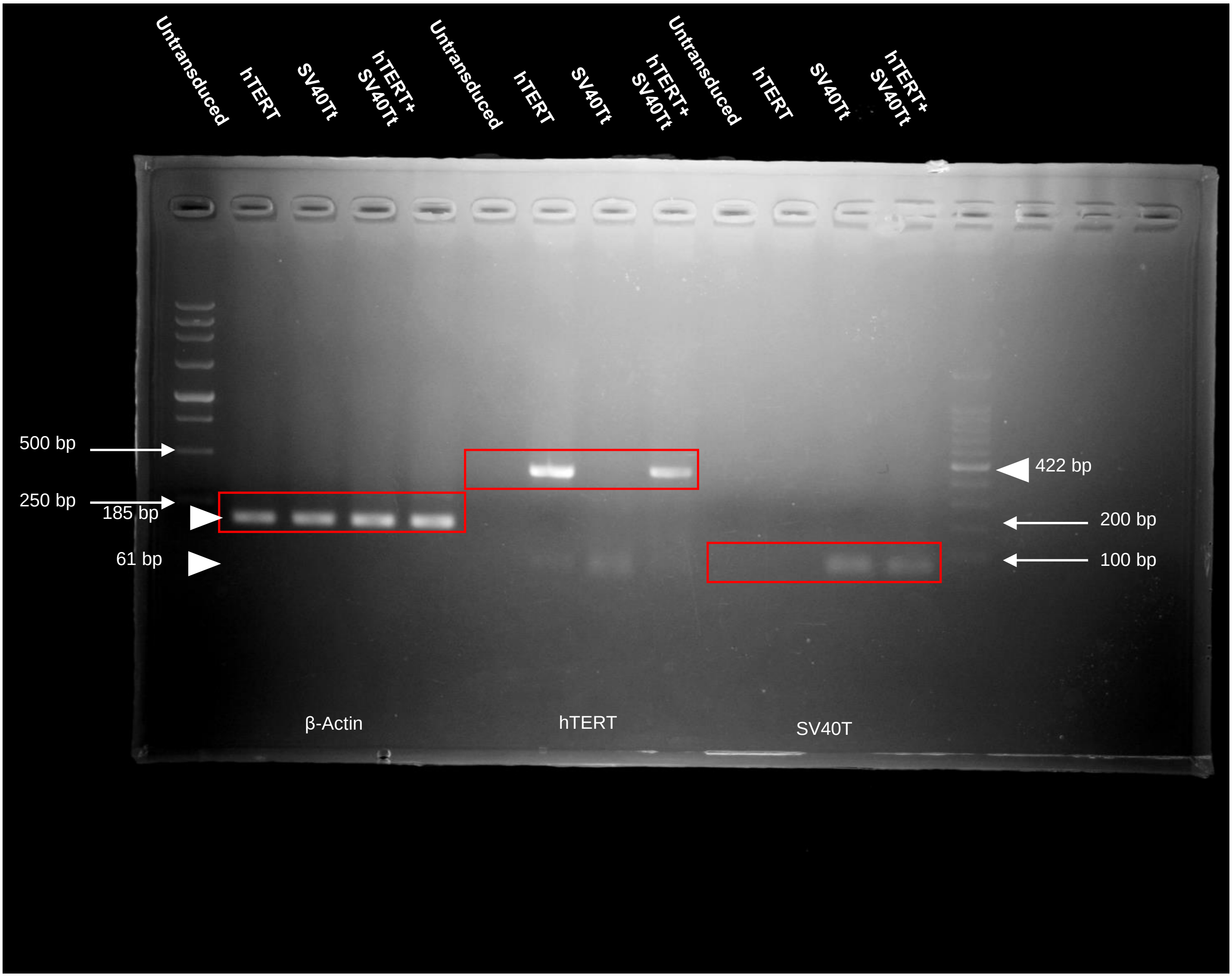

Supplementary Figure S8. Original image in Supplementary Figure S2a.
