# Supplementary material for: Establishment and characterization of immortalized sweat gland myoepithelial cells
Source: Sci Rep. 2022 Jan 7;12:7. doi: 10.1038/s41598-021-03991-5 (PMC8741770; doi:10.1038/s41598-021-03991-5)
Supplement: Supplementary file 2 — Supplementary Tables. [file 41598_2021_3991_MOESM2_ESM.pdf]

Title: Establishment and characterization of immortalized sweat gland myoepithelial cells

Authors and affiliations: Tomohisa Hayakawa[1], Fumitaka Fujita[1,2,*], Fumihiro Okada[1,2], Kiyotoshi Sekiguchi[3,*]

[1]Laboratory of Advanced Cosmetic Science, Graduate School of Pharmaceutical Sciences, Osaka University, 1-6 Yamadaoka, Suita Osaka *565-0871*, Japan

[2]Fundamental Research Institute, Mandom Corporation, Osaka, Japan

[3]Division of Matrixome Research and Application, Institute for Protein Research, Osaka University, 3-2 Yamadaoka, Suita Osaka *565-0871*, Japan

* Corresponding author

E-mail: sekiguch@protein.osaka-u.ac.jp; fujita-f@phs.osaka-u.ac.jp

Supplementary Table S1. List of components of spheroid culture medium.

Product name	Final concentration	Source	Cat. no.
MammoCult Human medium Kit	Base medium	Stemcell Technologies, Vancouver, BC, CA	ST-05620
Recombinant Human EGF	10 ng/mL	PeproTech, Rocky Hill, NJ	AF-100-15
Human basic FGF	10 ng/mL	Stemcell Technologies	ST-78003
0.2% heparin solution	4 μg/mL	Stemcell Technologies	ST-07980
Hydrocortisone 21-hemisuccinate sodium salt	0.5 μg/mL	Sigma-Aldrich, St. Louis, MO	H2270
Matrigel Growth Factor Reduced	2% (v/v)	BD Biosciences, San Jose, CA	354230
Antibiotic-antimycotic	X100	Gibco, Waltham, MA	15240062

Supplementary Table S2. List of antibodies used in western blot (WB) and immunofluorescence staining (IF).

Type	Target	Host	Conjugation	Source	Cat. no.	Dilution	
						WB	IF
1st antibody	Keratin 8	Mouse	-	Progen, Heidelberg, Germany	61038	-	1:200
1st antibody	Keratin 8	Rabbit	-	Abcam, Cambridge, UK	ab53280	1:1000	-
1st antibody	α-SMA	Rabbit	-	Abcam	ab5694	-	1:200
1st antibody	Integrin β1	Mouse	-	Abcam	ab3167	-	1:200
1st antibody	Keratin 14	Mouse	-	Abcam	ab7800	-	1:200
1st antibody	GAPDH	Mouse	-	Millipore, Bedford, MA	MAB374	1:1000	-
2nd antibody	Rabbit	Goat	Alexa Fluor 594	Thermo Fisher Scientific, Waltham, MA	A-11037	-	1:1000
2nd antibody	Mouse	Donkey	Alexa Fluor 488	Thermo Fisher Scientific	A-21202	-	1:1000
2nd antibody	Rabbit	Goat	HRP	Bio-Rad Laboratories, Hercules, CA	1706515	1:1000	-
2nd antibody	Mouse	Goat	HRP	Bio-Rad Laboratories	1706516	1:1000	-

Supplementary Table S3. Sequences of primer pairs used for qRT-PCR and PCR experiments.

Type	Gene		Sequence (5' to 3')
qRT-PCR	18S rRNA	Forward primer	CGGCTACCACATCCAAGGAA
		Reverse primer	AGCTGGAATTACCGCGGC
qRT-PCR	Keratin 18	Forward primer	CCCTGCTGAACATCAAGGTCAA
		Reverse primer	GCTGTCCAAGGCATCACCAA
PCR	hTERT	Forward primer	GAAGGCGTCTGGGATGCGAA
		Reverse primer	GAGTAGAGGAAGTGCTTGGT
PCR	SV40	Forward primer	GACTCAGGGCATGAAACAGG
		Reverse primer	ACTGAGGGGCCTGAAATGA
PCR	β-Actin	Forward primer	TGGCACCCAGCACAATGAA
		Reverse primer	CTAAGTCATAGTCCGCCTAGAAGCA

Supplementary Table S4. List of compounds used in cell culture for the differentiation of immortalized myoepithelial cells.

Product Name	Cat. no.	Target	Concentration
1-Azakenpaullone	S7193	GSK-3	5 µM
Amcasertib (BBI503)	S8572	Stemness kinase	10 µM
APTSTAT3-9R	S8197	STAT	10 µM
AR-A014418	S7435	GSK-3	500 nM
Astragaloside A	S2415	Calcium Channel	100 nM
AT9283	S1134	Aurora Kinase,Bcr-Abl,JAK	2 µM
Avagacestat (BMS-708163)	S1262	Beta Amyloid,Gamma-secretase	10 µM
AZD1480	S2162	JAK	10 µM
AZD2858	S7253	GSK-3	2 µM
BIO	S7198	GSK-3	10 µM
Bisindolylmaleimide I (GF109203X)	S7208	PKC	200 nM
Bisindolylmaleimide IX (Ro 31-8220 Mesylate)	S7207	PKC	1 µM
BMS-833923	S7138	Hedgehog/Smoothened	10 µM
CEP-33779	S2806	JAK	10 µM
CHIR-98014	S2745	GSK-3	500 nM
CP21R7 (CP21)	S7954	Wnt/beta-catenin	1 µM
Crenigacestat (LY3039478)	S7169	Gamma-secretase	10 µM
DAPT (GSI-IX)	S2215	Beta Amyloid,Gamma-secretase	10 µM
Decernotinib (VX-509)	S7541	JAK	4 µM
Dibenzazepine (YO-01027)	S2711	Gamma-secretase	1 µM
Enzastaurin (LY317615)	S1055	PKC	1 µM
Fasudil (HA-1077) HCl	S1573	Autophagy,ROCK	1 µM
Fedratinib (SAR302503, TG101348)	S2736	JAK	3 µM
FH535	S7484	PPAR,Wnt/beta-catenin	10 µM
Filgotinib (GLPG0634)	S7605	JAK	10 µM
FR 180204	S7524	ERK	10 µM
Galunisertib (LY2157299)	S2230	TGF-beta/Smad	10 µM
Gandotinib (LY2784544)	S2179	JAK	10 µM
GANT61	S8075	Gli	5 µM
GNF-5	S7526	Bcr-Abl	10 µM
Go 6983	S2911	PKC	10 µM
GSK429286A	S1474	ROCK	10 µM
GW788388	S2750	TGF-beta/Smad	100 nM
Hesperetin	S2308	TGF-beta/Smad,Histamine Receptor	5 µM
HO-3867	S7501	STAT	5 µM
IC261	S8237	Casein Kinase	10 µM
ICG-001	S2662	Wnt/beta-catenin	250 nM
IM-12	S7566	GSK-3	5 µM
Indirubin	S2386	GSK-3	500 nM
IWP-L6	S7301	Wnt/beta-catenin	3 µM
IWR-1-endo	S7086	Wnt/beta-catenin	10 µM
Jervine	S4747	Hedgehog/Smoothened	500 nM
K02288	S7359	TGF-beta/Smad	20 nM
KY02111	S7096	Wnt/beta-catenin	500 nM
KYA1797K	S8327	Wnt/beta-catenin	50 nM
LDN-193189 2HCl	S7507	TGF-beta/Smad	1 µM
LF3	S8474	Wnt/beta-catenin	10 µM
LGK-974	S7143	Wnt/beta-catenin	500 nM
LY2811376	S1528	BACE,Beta Amyloid	500 nM
LY411575	S2714	Gamma-secretase	100 nM
Methyl Vanillate	S3630	Wnt/beta-catenin	1 µM
Mivebresib(ABBV-075)	S8400	Epigenetic Reader Domain	10 µM
MK-0752	S2660	Beta Amyloid,Gamma-secretase	10 µM
MK-4101	S8200	Hedgehog/Smoothened	10 µM
NCB-0846	S8392	Wnt/beta-catenin	1 µM
NVP-BSK805 2HCl	S2686	JAK	300 nM
O4I2	S7944	Oct3/4	1 µM
OAC1	S7217	Oct3/4	300 nM
Oclacitinib maleate	S8195	JAK	10 µM
Pacritinib (SB1518)	S8057	FLT3,JAK	10 µM
PD173955	S7269	Bcr-Abl	2.5 µM
PF-5274857	S2777	Hedgehog/Smoothened	1 µM
Pirfenidone	S2907	TGF-beta/Smad	10 µM
PRI-724	S8262	Wnt/beta-catenin	10 µM
RepSox	S7223	TGF-beta/Smad	10 µM
RKI-1447	S7195	ROCK	10 µM
RO4929097	S1575	Beta Amyloid,Gamma-secretase	100 nM
SANT-1	S7092	Hedgehog/Smoothened	10 nM

Product Name	Cat. no.	Target	Concentration
SB216763	S1075	GSK-3	100 nM
SB415286	S2729	GSK-3	10 μM
SB431542	S1067	TGF-beta/Smad	5 μM
SB505124	S2186	TGF-beta/Smad	10 μM
SB525334	S1476	TGF-beta/Smad	1 μM
SCH772984	S7101	ERK	1 μM
Semagacestat (LY450139)	S1594	Gamma-secretase	10 μM
SH-4-54	S7337	STAT	10 μM
Silmitasertib (CX-4945))	S2248	Casein Kinase	1 μM
SKL2001	S8320	Wnt/beta-catenin	10 μM
Sonidegib (Erismodegib, NVP-LDE225)	S2151	Hedgehog/Smoothened	10 μM
Sotrastaurin	S2791	PKC	1 μM
Taladegib (LY2940680)	S2157	Hedgehog,Hedgehog/Smoothened	10 μM
TG101209	S2692	c-RET,FLT3,JAK	10 μM
Theophylline	S1621	TGF-beta/Smad	1 μM
Thiazovivin	S1459	ROCK	10 μM
TWS119	S1590	GSK-3	10 μM
Vismodegib (GDC-0449)	S1082	Hedgehog/Smoothened	2.5 μM
VX-11e	S7709	ERK	50 nM
WHI-P154	S2867	EGFR,JAK	5 μM
WIKI4	S7490	Wnt/beta-catenin	10 μM
Wnt agonist 1	S8178	Wnt/beta-catenin	10 μM
Wnt-C59 (C59)	S7037	Wnt/beta-catenin	5 μM
XAV-939	S1180	Wnt/beta-catenin	1 μM
XL019	S7036	JAK	10 μM
XMD8-92	S7525	ERK	10 μM
XMU-MP-1	S8334	Hippo pathway	10 μM
Y-27632 2HCl	S1049	Autophagy,ROCK	10 μM
ZM 39923 HCl	S8004	JAK	1 μM
β-Glycerophosphate sodium salt hydrate	S3620	phosphatase	1 μM

Source: Selleck Chemicals
